# Transcription Factor Rv1453 Contributes to Multidrug Sensitivity of *Mycobacterium tuberculosis*: A Potential Role for Lipid Metabolism

**DOI:** 10.3390/microorganisms14102376

**Published:** 2026-10-16

**Authors:** Lei Zhang, Chennan Liu, Xiaodong Li, Bin Wang, Jinghua Shi, Zimo Wang, Yu Lu

**Affiliations:** Department of Pharmacology, Beijing Key Laboratory of Drug Resistance Tuberculosis Research, Beijing Chest Hospital, Capital Medical University, Beijing Tuberculosis and Thoracic Tumor Research Institute, Beijing 101149, China

**Keywords:** *Mycobacterium tuberculosis*, transcription factor, Rv1453, cell wall, lipid metabolism, reduced multidrug susceptibility

## Abstract

The growing prevalence of multidrug-resistant *Mycobacterium tuberculosis* complicates tuberculosis control efforts. We discovered that Rv1453, as a new transcription factor, is crucial for lipid metabolism in bacteria. Rv1453 suppresses expression of genes related to fatty acid β-oxidation (*fadD18*, *fadE5*, *fadE12*, *fadE13*, *fadE18*, *fadE30*, *fadE31*, *echA7*) and genes for lipid synthesis (*accA2*, *accD2*). Furthermore, acyl-Coenzyme A influences signals regulated by Rv1453. Removing Rv1453 boosts expression of these genes, alters cell wall lipid composition, changes colony morphology, thickens the cell wall, and contributes to the reduced multidrug susceptibility. These findings point to an association between Rv1453-mediated lipid metabolism and multidrug sensitivity in *Mycobacterium tuberculosis*.

## 1. Introduction

Tuberculosis (TB) caused by *Mycobacterium tuberculosis* (*Mtb*) has been a longstanding global health issue. In 2022, 7.5 million new TB cases were reported, with 1.30 million deaths, including those with HIV. Multidrug-resistant TB (MDR/RR-TB) affected 3.3% of new cases and 17% of previously treated cases [1]. Understanding MDR-TB causes is crucial for effective public health strategies. Reduced multidrug susceptibility in *Mtb* arises from various factors, including rapid changes in gene expression under drug pressure, leading to a tolerant phenotype. The *Mtb* genome contains about 214 transcription factors that regulate gene expression throughout its life cycle [2,3]. Transcriptional repressors, such as ferric uptake regulators A (FurA), Rv1909c, Rv0678, and Rv1830, contribute to *Mtb*’s resistance to drugs like isoniazid (INH), rifampin (RFP), clofazimine (CFZ), and bedaquiline (BDQ) [4,5,6,7,8,9]. Specifically, *Rv0678* is identified as a BDQ-resistance-associated gene and regulates the MmpL5-MmpS5 efflux pumps system by binding to its promoter region, inhibiting gene expression, and drug efflux MmpL5 and MmpS5 constitute RND-type membrane transport efflux pumps. The *mmpL5* and *mmpS5* genes, located in the same operon, are regulated by the transcription factor *Rv0678*, which resides downstream of the mmpS5-mmpL5 operon. The Rv0678 protein binds to the promoter of the intergenic region between *Rv0678* and *MmpS5*, preventing RNA polymerase from accessing the transcription start site and thereby inhibiting the expression of mmpS5 and mmpL5, which suppresses drug efflux by the MmpS5-MmpL5 system [10]. Although the regulatory mechanisms of transcription factors are recognized as key determinants of reduced multidrug susceptibility in *Mtb*, the structural and functional characteristics of numerous transcription factors remain elusive.

Our previous studies [11,12] have shown that the transcriptional regulatory protein encoded by the *Rv1453* can bind to the genomic region encompassing the RNA polymerase binding site of *qor* (*Rv1454c*, which encodes quinone oxidoreductase), thereby inhibiting its transcription. Notably, we observe that the *Rv1453* gene knockout strain exhibited varying degrees of sensitivity to levofloxacin (LFX) and other agents, changes that were not attributable to alterations in the expression levels of the *qor* gene. The Rv1453 protein contains a classical helix-turn-helix (HTH) domain that facilitates DNA binding, indicating the potential presence of additional target genes beyond *qor*. In this study, we further explored the regulatory scope of Rv1453 and found that it directly modulates a substantial number of genes involved in the lipid metabolism of *Mtb*. The deletion of Rv1453 resulted in disrupted mycobacterial lipid homeostasis, general alterations in lipid content, changes in colony morphology, cell wall thickening, and reduced susceptibility to multiple drugs. This study identifies Rv1453 as a novel regulator of mycobacterial lipid metabolism. It provides insights into the association between Rv1453 deletion-related lipid metabolic alterations and reduced multidrug susceptibility in *Mtb*.

## 2. Materials and Methods

### 2.1. Bacterial Strains and Culture Conditions

The *E. coli* DH5α and *E. coli* BL21 (DE3) strains were grown in Luria–Bertani medium. The *Mtb* H37Rv, *Δ1453*, *Δ1453*/*C* strains were grown in 7H9 liquid medium supplemented with 0.2% glycerin, 0.05% Tween 80, and 10% oleic acid-albumin-dextrose-catalase (OADC) at 37 °C, or on 7H10 solid medium with 0.5% glycerin and 10% OADC at 37 °C. In the carbon-use experiment, the *Mtb* strains were cultured in carbon-defined minimal medium, which consists of modified Sauton’s medium [13,14]. Then 0.2% glucose and 0.2% glycerol, 0.02% sodium oleate (with 1% fatty acid-free bovine serum albumin [BSA]), 0.2% sodium valerate, or 0.1 mM cholesterol can be added as the sole carbon source. For the growth curve experiment [15], the *Mtb* strains were pre-cultured to OD600 of approximately 1.0. Subsequently, it was washed with 1 × PBS and then re-suspended in each medium to achieve an OD600 of around 0.01. Following this, a volume of 20 mL medium was added to a 60 mL cell culture vial and Colony-Forming Unit (CFU) counts were measured at successive time points. Statistical analysis was performed only for day-28 data. One-way ANOVA followed by Tukey’s post hoc test was used to evaluate differences among experimental strains.

### 2.2. Minimum Inhibitory Concentration (MIC) Determination

MIC determination was performed using the Microplate AlamarBlue assay (MABA) as described in our previous publication [11]. The MIC was defined as the lowest concentration that elicited more than 90% reduction in fluorescence compared to drug-free controls, or prevented the color change from blue to pink. LFX, moxifloxacin (MFX), pretomanid (PA-824), and BDQ ranged from 5 to 0.01 μg/mL, and ethambutol (EMB) and linezolid (LZD) ranges from 25 to 0.049 μg/mL. The concentration of delamanid (DLM) ranges from 78 to 0.152 ng/mL. All drugs were tested using a two-fold dilution method.

### 2.3. Transcriptional Profiling

Total RNA isolation and sample preparation for RNA-Seq were performed as previously reported [12]. The *Mtb* H37Rv and *Δ1453* strains were cultured in 7H9 medium until the logarithmic growth phase, which took approximately 14 days. Then 2 × 10^8^ cells were collected for total RNA extraction using the TruSeq^®^ Stranded Total RNA Sample Preparation kit (Illumina, San Diego, CA, USA). Ribosomal RNA was removed from total RNA using Ribo-Zero rRNA removal beads. The mRNA was fragmented then converted to the cDNA using the same method as previously reported [16]. The final cDNA library was purified and enriched by PCR. The library construction and sequencing were performed at Shanghai Biotechnology Corporation. RNA integrity was assessed using an Agilent Bioanalyzer 2100 (Agilent technologies, Santa Clara, CA, USA). Sequencing was performed on the Illumina HiSeq 2500 platform (Illumina, San Diego, CA, USA), with approximately 20 million paired-end reads per sample. Raw reads were quality-filtered, mapped to the *M. tuberculosis* H37Rv reference genome (NC_000962.3) using Bowtie2 (version 2.2.0.5), with a mapping rate > 90%, and gene counts were generated with HTSeq. Normalization and differential expression analysis were performed with DESeq2. Multiple-testing correction was performed using the Benjamini-Hochberg procedure to control the false discovery rate (FDR). Differentially expressed genes were identified with adjusted *p* value [*p*adj] of <0.05 and |log_2_fold change| > 1.

### 2.4. Expression and Purification of Recombinant Protein

The plasmid pET30a was used for the expression of Rv1453 in *E.coli* BL21(DE3) [12]. Briefly, after the cells had 4 h of growth, Rv1453 protein was induced by the addition of isopropyl–D-1-thiogalactopyranoside (IPTG) at a final concentration of 0.5 mM. Under this condition, Rv1453 was expressed as inclusion bodies. Capturem™ His-Tagged Purification Kit (TaKaRa, Kyoto, Japan) was used, and the protein was purified with 8M urea under denaturing conditions. To obtain functionally active protein for subsequent assays, gradient urea dialysis was performed for *in vitro* refolding. Briefly, the denatured protein solution was sequentially dialyzed against buffer containing gradually decreased urea concentrations (6 M, 4 M, 2 M, and 0 M urea) at 4 °C to allow gradual and correct protein refolding. After dialysis, the refolded protein was centrifuged to remove precipitates, and the supernatant containing soluble refolded protein was collected. Protein purity was routinely examined by SDS-PAGE (Supplementary Figure S6). The concentration of Rv1453 protein was tested by the BCA assay (Solarbio, Beijing, China).

### 2.5. Electrophoretic Mobility Shift Assay (EMSA)

The promoter regions of *Rv0976c*, *fadD18*, *fadE5*, *fadE18*, *fadE30*, and *fadE31* were amplified from *Mtb* genomic DNA using specific primers and purified with a DNA purification kit (Takara). Short DNA segments (motif) were synthesized by RuiBiotech (Beijing, China) *in vitro* and directly annealed before use. The EMSA buffer and procedure were based on previous reports [12,17]. For promoter regions of *Rv0976c*, *fadD18*, *fadE5*, *fadE18*, *fadE30*, and *fadE31*, the binding reaction mixture (10×) contained 100 mmol/L Tris-HCl, 500 mmol/L KCl, 10 mmol/L DTT, 50% glycerol, 10 mmol/L EDTA. The Rv1453 and promoter regions DNA segment were incubated at 37 °C for 1.5 h. Then, the samples were separated by the native PAGE gel in an ice bath containing 1 × Tris-borate-EDTA at 150 V for 1 h. The native PAGE gel was stained with SYBR Green I dye for about 15 min and placed in an ultraviolet analyzer to observe the bands. Meanwhile, the 5′-end biotin-labeled DNA was incubated with Rv1453 at 37 °C for 1.5 h in this study. Electrophoretic mobility shift assays (EMSAs) were carried out according to manufacturer’s protocol for the LightShift™ Chemiluminescent EMSA Kit (Thermo Fisher, Waltham, MA, USA). The binding reaction mixture contained 10 mmol/L Tris-HCl, 50 mmol/L KCl, 1 mmol/L DTT, 5% glycerol, 1 mmol/L EDTA, and 50 μg/mL poly (dI-dC). Images of gels were obtained using a Typhoon Scanner (GE Healthcare, Chicago, IL, USA).

### 2.6. Analysis of β-Galactosidase (lacZ) Activity

lacZ activity experiments were performed in *Mtb*. A series of operon-lacZ fusion plasmids derived from pMV261 was constructed. The recombinant plasmids were then transformed into H37Rv or *Δ1453* strains to obtain corresponding reporter strains. All strains were grown in 7H9 at 37 °C to an OD600 of 0.5, and the cells were harvested to measure lacZ activity as described previously [18]. Mann–Whitney U test was applied to calculate the statistical significance (*p* value).

### 2.7. Fluorescence Polarization Assay (FPA)

The FITC fluorescence-labeled double-strand DNA of sequence motif was chemically synthesized by RuiBiotech (Beijing, China). The DNA fragments (5 nM) were incubated at 37 °C for 30 min with purified Rv1453. FPAs were performed using a plate reader (Spark; Tecan, Grödig, Austria) equipped with an excitation filter of 488 nm and an emission filter of 525 nm.

### 2.8. Lipidomics

The lipidomics were performed as previously reported [19]. Briefly, the *Mtb* cells were grown in 7H9 medium and harvested at mid-logarithmic phase for lipidomic analysis. Lipid extraction, ultra-performance liquid chromatography, mass spectrometry, and lipid structural analyses were performed at Biotree (Shanghai, China). The samples were dried, reconstituted in 50% methanol/dichloromethane, vortexed, and sonicated. After centrifugation, the supernatant was analyzed by LC-MS/MS using a Vanquish UHPLC system and a Kinetex C18 column. The mobile phases consisted of water/acetonitrile/ammonium formate and acetonitrile/isopropanol/ammonium formate. The QE mass spectrometer was operated in data-dependent acquisition mode. Raw data were converted to mzXML format, and peak detection, alignment, and lipid identification were performed using XCMS and LipidBlast library. Unpaired two-tailed *t* test (*t*-test) was applied to calculate the statistical significance (*p* value) and fold change in the metabolites between the means of the two groups.

### 2.9. Transmission Electron Microscopy (TEM)

TEM was performed as previously described [20]. Briefly, the cells were fixed, gradually dehydrated, and polymerized using Epon 812 resin (SPI Supplies, West Chester, PA, USA); 70~90 nm sections were cut using an Leica UC7 ultramicrotome (Leica Microsystems, Wetzlar, Germany) and subsequently stained using uranyl acetate and lead citrate for visualization under a HITACHI HT7800 transmission electron microscope (Hitachi High-Tech, Tokyo, Japan).

### 2.10. Cell-Wall Permeability Assay

Cell-wall permeability of *Mtb* strains was assayed by measuring the intracellular accumulation of ethidium bromide (Etbr) as previously reported [21]. Cells exponentially growing in 7H9 with glycerol and OADC were washed and resuspended in potassium phosphate buffer (pH 7) to an OD600 of ~0.5. After addition of Etbr (2 μM), the fluorescence was monitored over time using a plate reader (Spark; Tecan) equipped with an excitation filter of 540/25 nm and an emission filter of 580/20 nm.

### 2.11. Macrophage Infection Study

J774A.1 (ATCC TIB-67, RRID: CVCL_0358) was obtained from the American Type Culture Collection (ATCC). J774A.1 cells were seeded at 4 × 10^5^ cells/well in a 24-well plate and incubated for 1 day before infection. Homogenized bacterial suspension at a multiplicity of infection (MOI) of 1, was transformed into adherent J774A.1 cells. After 2 h of infection, extracellular unbound bacteria were thoroughly removed by three washes with phosphate buffer saline (PBS). The infected macrophages were continuously incubated for 2 days under 5% CO_2_ at 37 °C.

For intracellular bacterial survival quantification, cell lysates were serially diluted 10-fold and plated onto 7H10-OADC agar plates. CFUs were enumerated after 28 days of incubation at 37 °C.

Cytotoxicity induced by different mycobacterial strains in J774A.1 macrophages was evaluated using a lactate dehydrogenase (LDH) release assay. The LDH cytotoxicity assay was performed using a commercial kit (Beyotime, Shanghai, China) strictly following the manufacturer’s protocols. One-way ANOVA test was applied to calculate the statistical significance (*p* value).

## 3. Results

### 3.1. Susceptibility of Mtb Strains to Anti-Tuberculosis Drugs

Using the MABA, we assessed the MIC values of various anti-tuberculosis drugs against the H37Rv, *Δ1453*, and *Δ1453/C* strains. The MIC results indicated that, compared to the H37Rv and the complemented *Δ1453/C* strains, the *Δ1453* strain exhibited diminished sensitivity to multiple anti-tuberculosis drugs, resulting in varying degrees of increased MIC values. These drugs included first-line anti-tuberculosis drugs, such as EMB, as well as LFX and MFX. Furthermore, novel anti-tuberculosis drugs such as PA-824, DLM, and BDQ also demonstrated reduced efficacy against the *Δ1453* strain. Notably, the *Δ1453* strain displayed significantly reduced sensitivity to LFX, LZD, and PA-824, with MIC values increasing by more than 10-fold compared to the H37Rv strain (Table 1). This suggests that the expression of the *Rv1453* gene is critical for the activity of anti-tuberculosis drugs. A previous study has demonstrated the overexpression of *qor* in the Rv1453-disrupted strain [12]. That study’s aim was to investigate whether the reduced sensitivity of the *Δ1453* strain to multiple drugs is associated with the overexpression of the *qor* gene. However, our findings indicate that overexpression of the *qor* gene does not diminish antibiotic susceptibility. In some instances, it actually increases susceptibility (Supplementary Table S1). This suggests that other target genes, but not the *qor* gene, are likely involved in mediating the observed changes in the *Δ1453* strain in relation to Rv1453.

**Figure 1:**
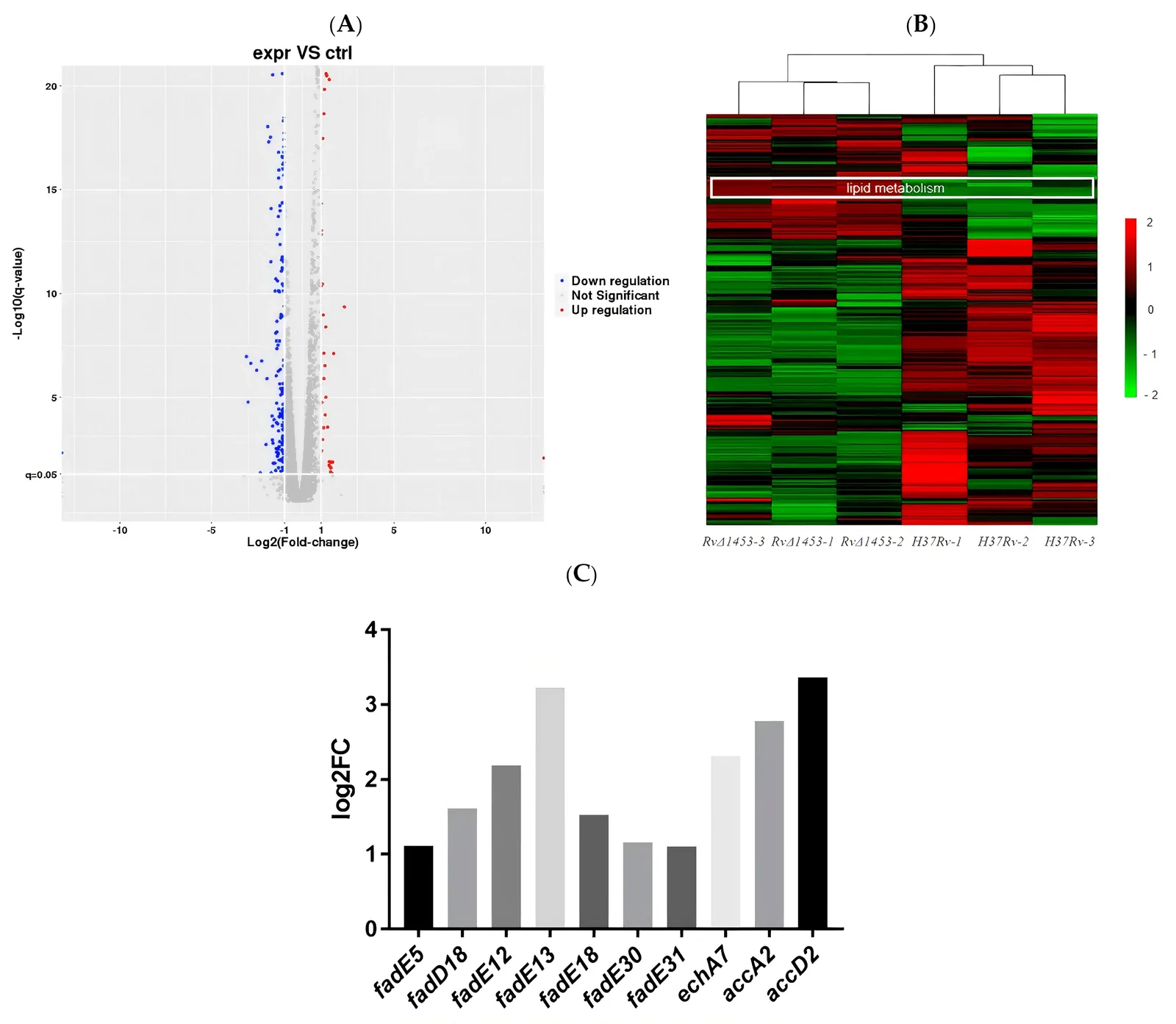
Transcriptomic assays of the effect of Rv1453 on *Mtb* gene expression. (**A**) Global expression levels of all genes differentially expressed in the transcriptome of Mtb *Δ1453* strain compared to the H37Rv strain. Note: Red spots indicate the genes that show upexpression (*p*adj < 0.05), blue dots indicate the genes that show downexpression (*p*adj < 0.05). (**B**) Transcriptome clustering analysis of *Δ1453* strains and H37Rv strains, *n* = 3. (**C**) Expression levels of lipid metabolism genes, *n* = 3.

To further examine the impact of Rv1453 on mycobacterial drug sensitivity, we conducted a comprehensive transcriptomic analysis of the H37Rv and *Δ1453* strains. In this plot, red dots denote genes that significantly up-regulated (2-fold change; adjusted *p* value [*p*adj] of <0.05), while blue dots indicate genes that are significantly down-regulated (2-fold change; *p*adj of <0.05) (Figure 1A). A heatmap was generated for three independent biological replicates of each sample, illustrating a set of up-regulated and down-regulated genes in the H37Rv and *Δ1453* strains (Figure 1B). The deletion of Rv1453 led to the identification of 240 DEGs, with 171 genes down-regulated and 69 genes up-regulated.

### 3.2. Rv1453 Negatively Regulates the Expression of Fatty Acid β-Oxidation Genes and Lipid Synthesis Genes

To further elucidate the regulatory mechanism of Rv1453 in bacterial lipid metabolism, the differentially expressed genes were subjected to enrichment and clustering analyses to identify the affected metabolic pathways utilizing the Gene Ontology (GO) and Kyoto Encyclopedia of Genes and Genomes (KEGG) pathway analyses. Notably, the *Δ1453* strain exhibited significant enrichment of 10 genes implicated in lipid metabolic pathways compared to the H37Rv strain (*p*adj < 0.05). Remarkably, two of the top six pathways were related to fatty acid metabolism, suggesting that Rv1453 plays a role in the regulation of fatty acid metabolism (Supplementary Figure S1A–D).

**Figure 2:**
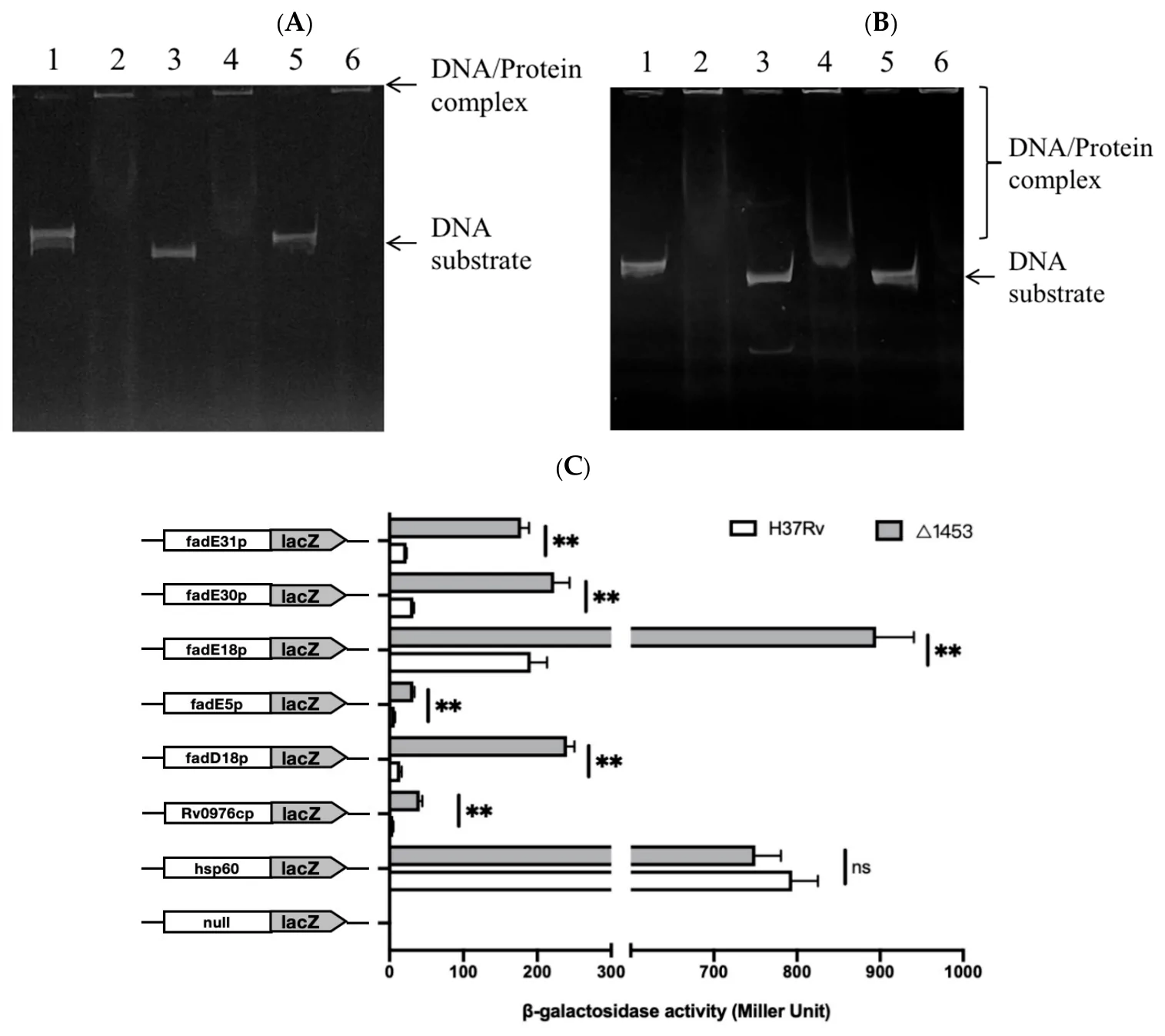
Rv1453 regulates the expression of lipid metabolism genes *in vitro* and *in* *vivo*. (**A**,**B**) EMSAs for DNA-binding activity of Rv1453 with the promoters of the gene operon (*fadE18p*, *fadE5p*, *fadD18p*, *Rv0976cp*, *fadE31p*, *fadE30p*). Note: (**A**) 1: *fadE18p*; 2: *fadE18p* + Rv1453; 3: *fadE5p*; 4: *fadE5p* + Rv1453; 5: *fadD18p*; 6: *fadD18p* + Rv1453; (**B**) 1: *Rv0976cp*; 2: *Rv0976cp* + Rv1453; 3: *fadE31p*; 4: *fadE31p* + Rv1453; 5: *fadE30p*; 6: *fadE30p* + Rv1453. (**C**) LacZ activity assays for the effect of Rv1453 on the expression of lipid metabolism genes. Note: LacZ activity is shown as Miller units in the right panel. *n* = 6, error bars indicate median (interquartile range). The significant differences were determined by Mann–Whitney U test (ns, no significance; ** *p* < 0.01).

A detailed analysis of gene localization revealed that the genes were part of distinct operons. But the genes *fadE12* (*Rv0972c*, acyl-CoA dehydrogenase FadE12), *fadE13* (*Rv0975c*, acyl-CoA dehydrogenase FadE13), *accA2* (*Rv0973c*, acetyl/propionyl-CoA carboxylase subunit alpha), *accD2* (*Rv0974c*, acetyl-/propionyl-CoA carboxylase subunit beta), and *echA7* (*Rv0971c*, enoyl-CoA hydratase EchA7) were identified as components of the same operon (Supplementary Figure S2). To ascertain whether Rv1453 could interact with these six operons and directly regulate the expression of associated genes, an *in* *vitro* EMSA was conducted. As depicted in Figure 2A,B (Supplementary Figure S3A,B), a progressive increase in the amount of shifted DNA was observed upon the addition of the Rv1453 protein to the reactions containing promoter DNA fragments. Subsequently, to further validate the influence of the Rv1453 regulator on the expression of lipid metabolism genes in *Mtb* *in vivo*, a series of promoter-*lacZ* fusion plasmids were constructed using *lacZ* as the reporter gene. As shown in Figure 2C, the hsp60 promoter significantly enhanced the expression of lacZ, resulting in elevated lacZ activity, which indicates the efficacy of the assay. The expression of lacZ under the promoters of lipid metabolism genes in H37Rv strains was comparatively lower than in *Δ1453* strains. Collectively, these findings suggest that Rv1453 acts as a negative regulator of lipid metabolism gene expression.

**Figure 3:**
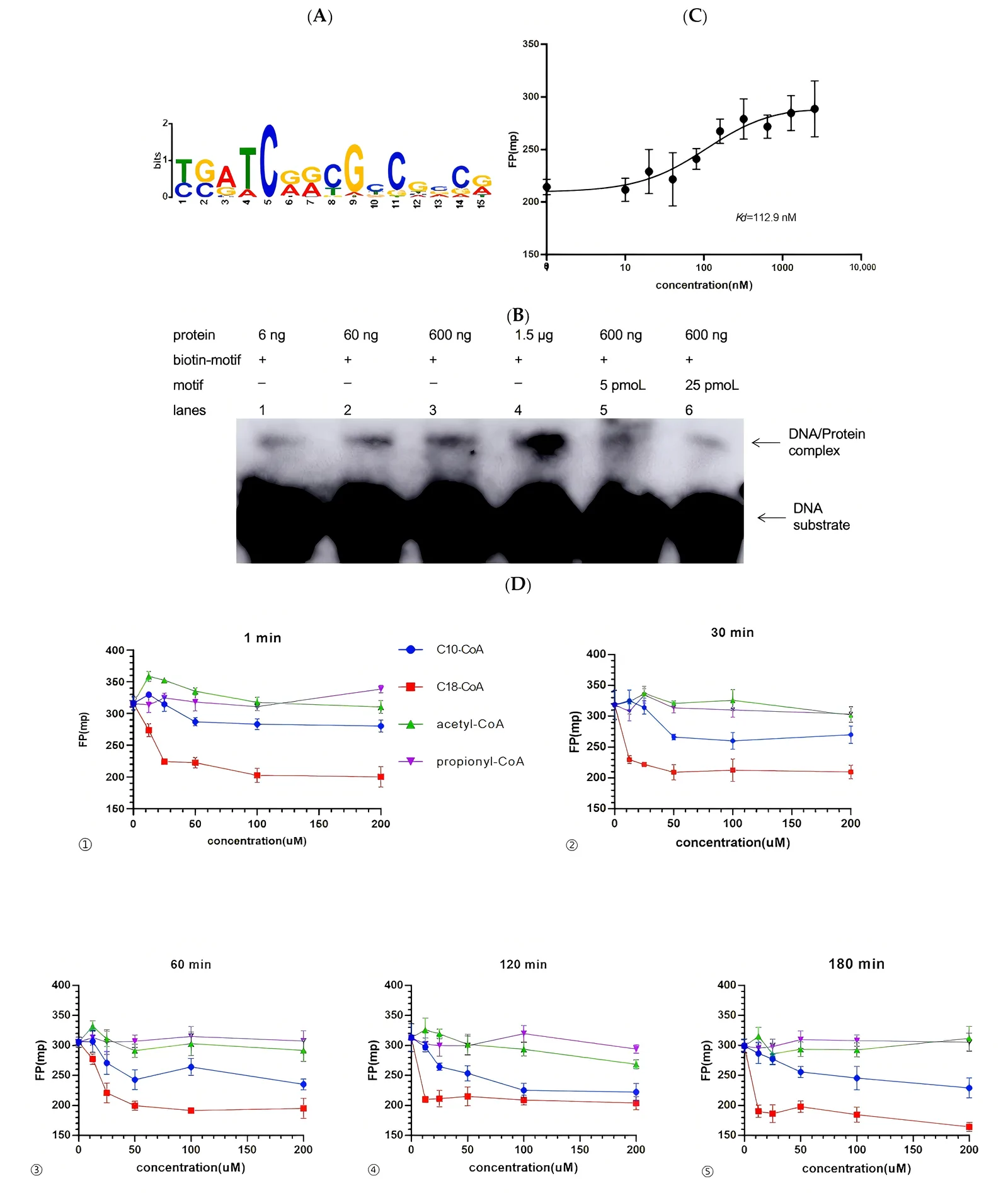
The motif sequence recognized by Rv1453 and the effect of various CoA on the DNA binding affinity of Rv1453. (**A**) Analysis of the conserved motif sequences specially recognized by Rv1453; (**B**) Competitive EMSA to determine the binding of Rv1453 to the motif sequence; (**C**) FPAs to determine the interaction between Rv1453 and the motif; (**D**) FPAs for the effect of C10-CoA, C18-CoA, acetyl-CoA, and propionyl-CoA on the DNA-binding affinity of Rv1453. Fluorescence polarization (FP, in mP) was measured at the indicated time points, 1 (①), 30 (②), 60 (③), 120 (④), and 180 (⑤) min after adding the specified CoAs to the reaction mixture. Data are presented as the means ± standard from three independent experiments.

Utilizing the binding sequences enriched in EMSA experiments, a conserved binding sequence motif for Rv1453 was identified using the MEME suite (Figure 3A). To assess Rv1453 binding, a 15-bp biotin-labeled motif was synthesized, and the Rv1453 protein from *Mtb* was employed in EMSA and competitive EMSA, which confirmed the binding of Rv1453 to the motif sequence. The mobility shift signal intensified with increasing concentrations of Rv1453 protein (Figure 3B, Lanes 1–4, Supplementary Figure S3C). To assess binding to this sequence motif, unlabeled motif-containing competitor DNA was added at 5 pmol and 25 pmol (Figure 3B, lanes 5–6), resulting in decreased intensity of the shifted band.

The binding affinity of the Rv1453 protein to this motif was assessed using FPAs. Upon DNA binding to the protein, there is a reduction in molecular freedom, resulting in an increased polarization value, which confirms the interaction of the Rv1453 protein and the motif (Figure 3C). The apparent dissociation constant (*K*_d_) for the interaction between Rv1453 and the motif was determined to be 112.9 nM through curve fitting. Collectively, these results indicate that in *Mtb*, Rv1453 acts as a negative regulator of the expression of a broad array of genes involved in fatty acid β-oxidation and lipid synthesis by targeting the promoters of gene clusters.

### 3.3. Acyl-CoAs Inhibit Rv1453 DNA Binding Affinity

Given that Rv1453 functions as a transcriptional repressor of lipid metabolism genes in *Mtb*, intermediate metabolites of lipid metabolism may influence the protein’s DNA-binding capability. Therefore, to identify the effector molecule(s) recognized by Rv1453 that modulate its transcriptional activity, we examined a set of compounds associated with mycobacterial lipid metabolism by analyzing their ability to modulate Rv1453’s binding to the motif. FPAs were conducted to evaluate the impact of decanoyl-CoA monohydrate (C10-CoA), stearoyl-CoA (C18-CoA), acetyl-CoA, and propionyl-CoA on the DNA-binding activity of Rv1453. As shown in Figure 3D, both C10-CoA and C18-CoA were found to inhibit the DNA-binding capacity of Rv1453, whereas acetyl-CoA and propionyl-CoA exhibited no such effect.

### 3.4. Rv1453 Inhibits Fatty Acid Accumulation in Mtb

**Figure 4:**
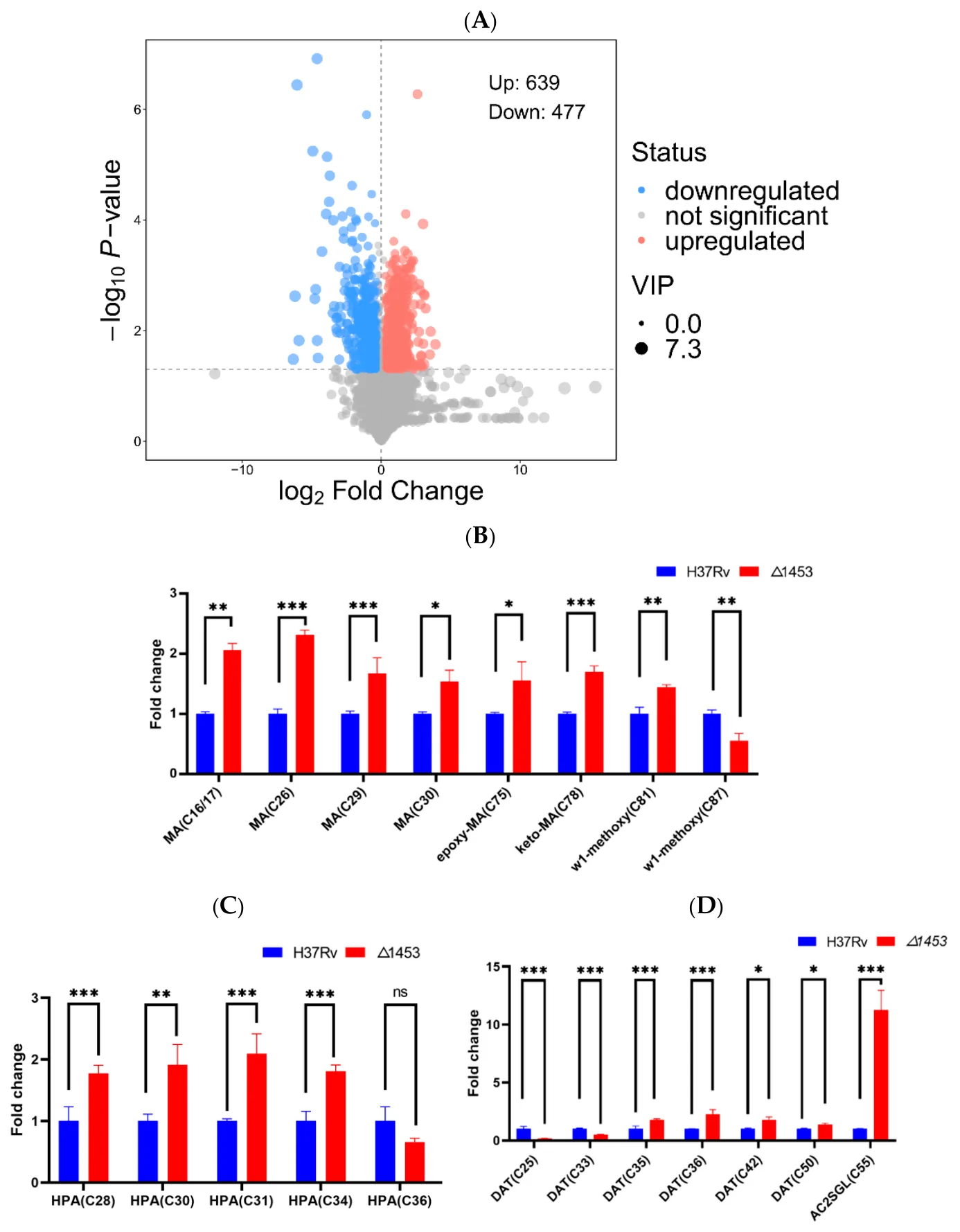
Comparative metabolic profiling assays for the effect of Rv1453 on the fatty acid levels of *Mtb*. (**A**) Volcano plot analysis of the differential lipid expression between the *Δ1453* and H37Rv strains detected by lipidomic assays. Increased abundance lipids are indicated by red spots. Decreased abundance and non-significantly changed lipid levels are indicated by blue and grey spots, respectively. (**B**) MAs were increased abundance in the cell wall of *Δ1453* strain compared to H37Rv strain. (**C**) HPAs were increased abundance in the cell wall of *Δ1453* strain compared to H37Rv strain. (**D**) DAT and Ac2SGL were increased abundance in the cell wall of *Δ1453* strain compared to H37Rv strain. Note: Data shown in (**B**–**D**) are mean ± SD (*n* = 3). * *p* < 0.05, ** *p* < 0.01, *** *p* < 0.001, not significant (ns), by unpaired two-tailed *t* test. Results with unadjusted *p* < 0.05 are presented as exploratory lipid changes without multiple-testing correction.

Rv1453 plays a crucial role in regulating the expression of genes associated with fatty acid β-oxidation and lipid synthesis, thereby facilitating the conversion of fatty acids into acetyl-CoA and propionyl-CoA. Subsequently, these metabolites enter the tricarboxylic acid cycle and the gluconeogenic pathway to produce energy and synthesize lipids, particularly those constituting the cell wall lipids. Consequently, we hypothesized that Rv1453 might influence the accumulation of cellular fatty acids in *Mtb*. To test this hypothesis, we conducted a lipidomic analysis to compare the relative lipid composition between the *Δ1453* and H37Rv strains. As shown in Figure 4A, 639 lipid species exhibited increased abundance (fold change > 1.2, unadjusted *p* < 0.05), whereas 477 displayed decreased abundance (fold change < 0.8, unadjusted *p* < 0.05). These exploratory observations are consistent with our data, indicating that Rv1453 negatively regulates the expression of genes involved in lipid metabolism.

Subsequently, we mapped the differential lipids against mycobacterial lipid databases (Mycomass, Mycomap), yielding 112 increased abundance and 108 decreased abundance lipid species (Table 2). We conducted a detailed analysis of the differentially expressed lipids within the cell wall lipidome. The analysis revealed that, compared to the H37Rv strain, the *Δ1453* strain exhibited elevated levels of branched acids. These included mycolic acid (MA) (C16/17), MA (C26), MA (C29), MA (C30), epoxy-MA (C75), keto-MA (C78), and methoxy-modified branched acid Methoxy-MA (C81), all of which are key constituents of cell wall lipids (Figure 4B). This exploratory observation suggests that loss of Rv1453 may favor MA biosynthesis in the *Mtb* cell wall. Methyl-branched fatty acids act as essential precursors for the biosynthesis of diacyltrehaloses (DAT), penta-acyltrehaloses (PAT), sulfolipid-1 (SL-1), and diacylated sulfoglycolipid (Ac2SGL) (Figure 4D). Moreover, long-chain methyl-branched fatty acids including hydroxyphthioceranic acid (HPA) (C28), HPA (C30), HPA (C31), and HPA (C34) displayed >1.5-fold higher abundance in the *Δ1453* strain cell wall compared with the H37Rv strain (Figure 4C). Of note, all lipidomic alterations were identified based on unadjusted *p* values; these results should be interpreted as exploratory rather than statistically validated findings.

### 3.5. Rv1453 Affects the Colony Phenotype, Cell Wall, and Growth of Mtb

**Figure 5:**
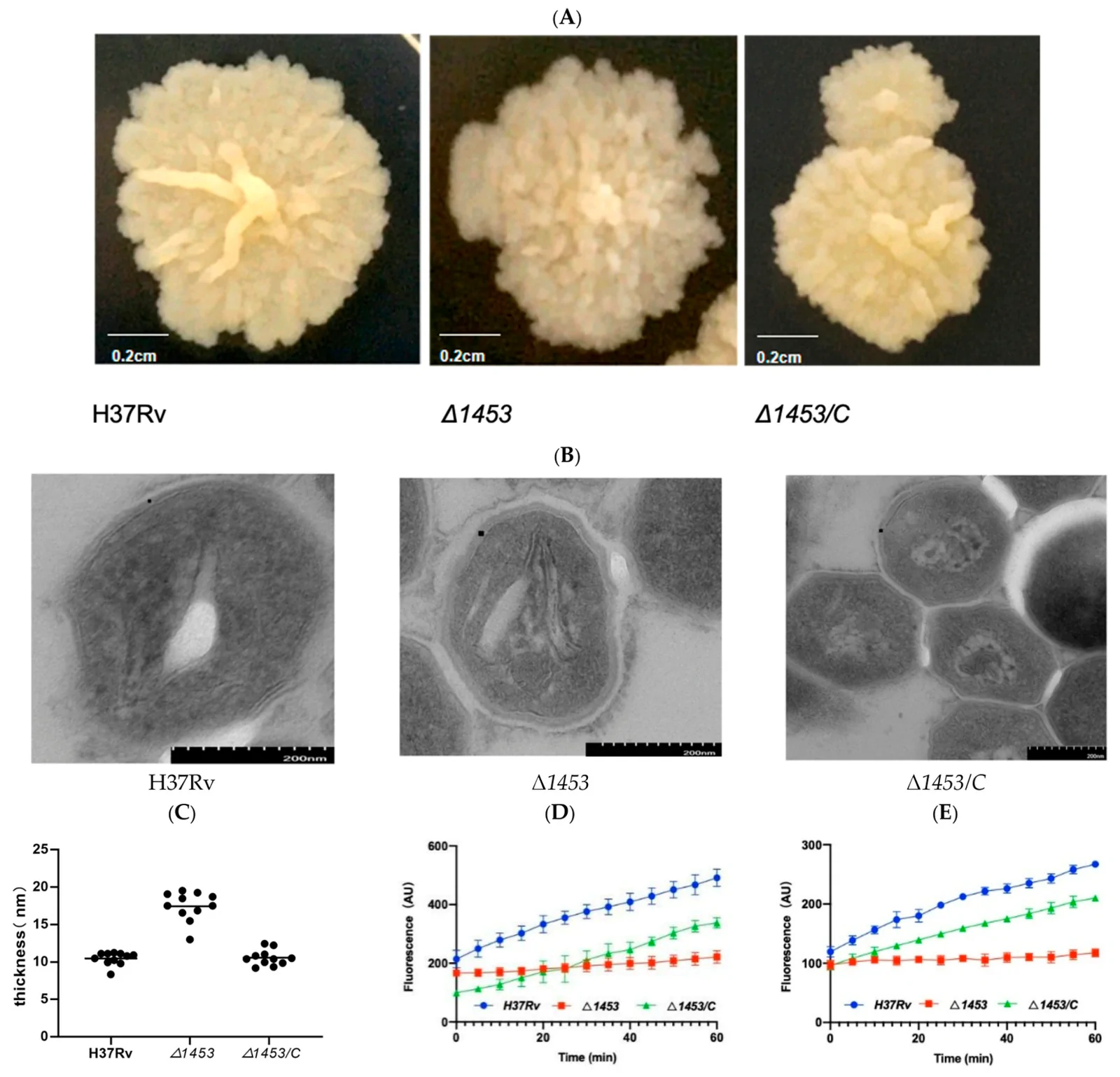
The impact of Rv1453 deletion in *Mtb*. (**A**) H37Rv, *Δ1453* and *Δ1453/C* forms of single colony morphology of *Mtb*. (**B**) TEM observation of the cell walls of each strain. The square labeled the cell wall of *Mtb*. (**C**) Statistical analysis of cell wall thickness of each strain. Note: *n* = 10 indicates ten individual bacterial cells sampled from a single biological preparation. (**D**) EtBr accumulation assay analysis of cell wall permeability of each strain without VP incubation. (**E**) EtBr accumulation assay analysis of cell wall permeability of each strain with 512 μM VP incubation.

We conducted separate plating of dilutions for the H37Rv, *Δ1453*, and *Δ1453/C* strains to observe the colony morphology of individual colonies. Our observations revealed that, compared to the H37Rv strain, the *Δ1453* strain exhibited a marked increase in surface folding and a rougher colony surface morphology (Figure 5A). Conversely, the complemented strain *Δ1453/C* showed colony morphology similar of the strain H37Rv. These findings suggest that the Rv1453 plays a regulatory role in the colony morphology of *Mtb*.

Subsequently, we evaluated the cell wall ultrastructure of the H37Rv, *Δ1453*, and *Δ1453/C* strains using TEM. The TEM analysis (Figure 5B,C, Supplementary Figure S4) revealed a tendency for thicker cell walls in the *Δ1453* strain relative to H37Rv and *Δ1453/C*, which may contribute to decreased cell wall permeability. To test this hypothesis, we conducted a cell permeability assay. Upon incubation with EtBr, the *Δ1453* strain exhibited a significant decrease in fluorescence compared to the H37Rv and the *Δ1453/C* (Figure 5D), confirming reduced cell wall permeability in the *Δ1453* strain. To examine the potential impact of efflux pumps on EtBr accumulation, the efflux pump inhibitor verapamil (VP) was utilized at a concentration of 512 μM in the experimental setup. The result (Figure 5E) indicated no significant alteration in the pattern of EtBr enrichment in these strains, suggesting that the decreased cell wall permeability observed in the *Δ1453* strain is not attributed to increased EtBr efflux.

**Figure 6:**
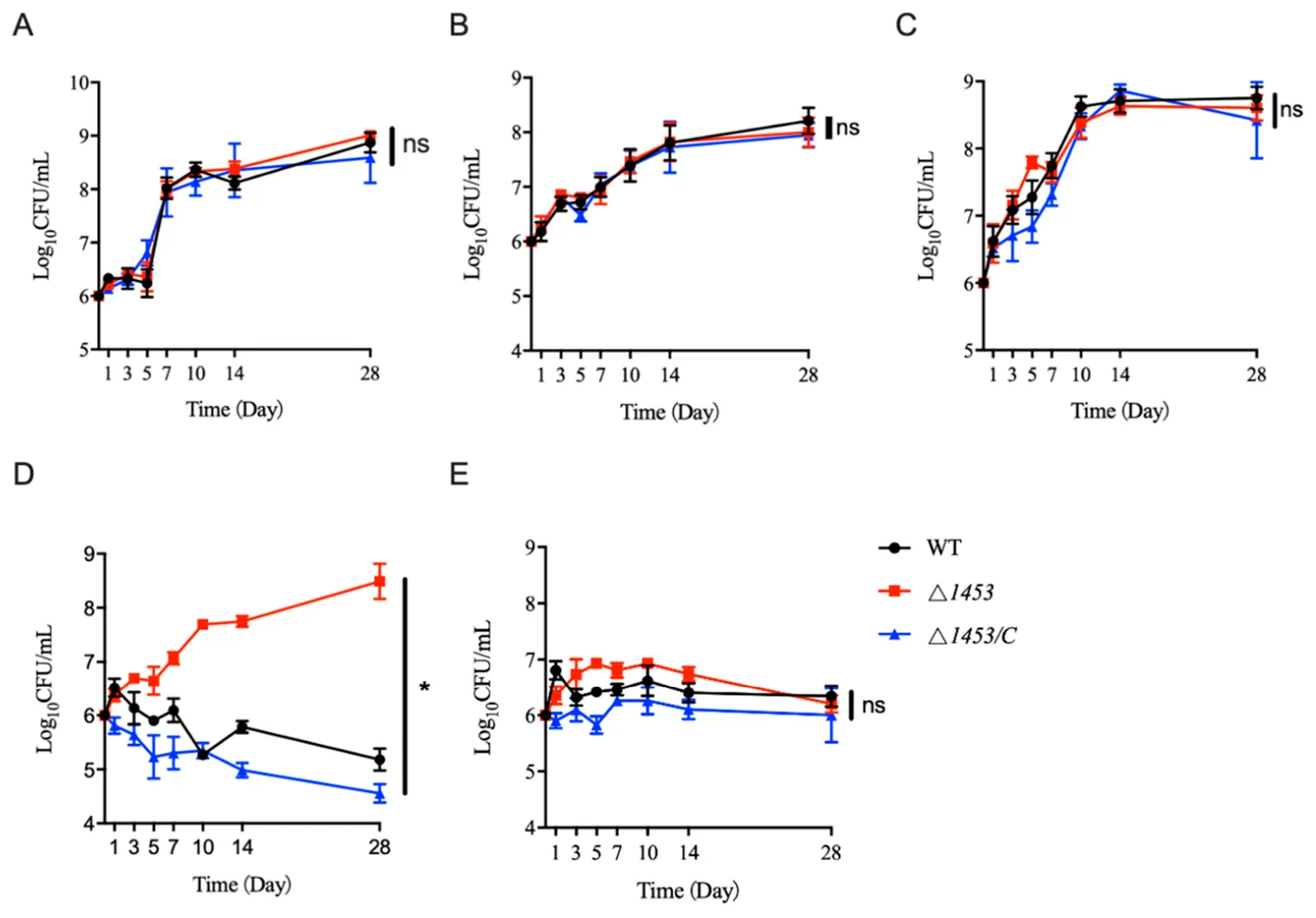
Growth curves of different strains of *Mtb* in media with different carbon sources. Note: H37Rv, *Δ1453*, and *Δ1453/C* were grown in 7H9 (**A**) or media with glucose and glycerol (**B**), oleic acid (**C**), sodium valerate (**D**), and cholesterol (**E**) as carbon sources. Data are the means ± standard errors from three experiments. * *p* < 0.05, statistical significance was determined using a one-way ANOVA test.

Given the alterations in colony phenotype and cell wall structure resulting from the deletion of Rv1453, which are closely associated with the lipid composition of *Mtb*, we investigated whether the loss of Rv1453 would enhance the growth of mycobacterial cells on fatty acids and related substrates. To assess this, we compared the growth of the H37Rv strain and the *Δ1453* strain in a carbon-defined minimal medium with various carbon sources (Figure 6). Both strains exhibited similar growth patterns when glucose, glycerol, oleic acid, cholesterol, or MB7H9 medium served as the sole carbon source (Figure 6A–C,E). However, the *Δ1453* strain unexpectedly demonstrated a growth advantage when sodium valerate was used as the sole carbon source (Figure 6D). This growth advantage was completely negated by the expression of Rv1453 in the *Δ1453* strain. Consequently, the *Δ1453* strain demonstrates an enhanced capacity to utilize sodium valerate as its sole carbon source.

### 3.6. Deletion of Rv1453 Increased Cell Cytotoxicity but Had No Effect on Intracellular Survivability

**Figure 7:**
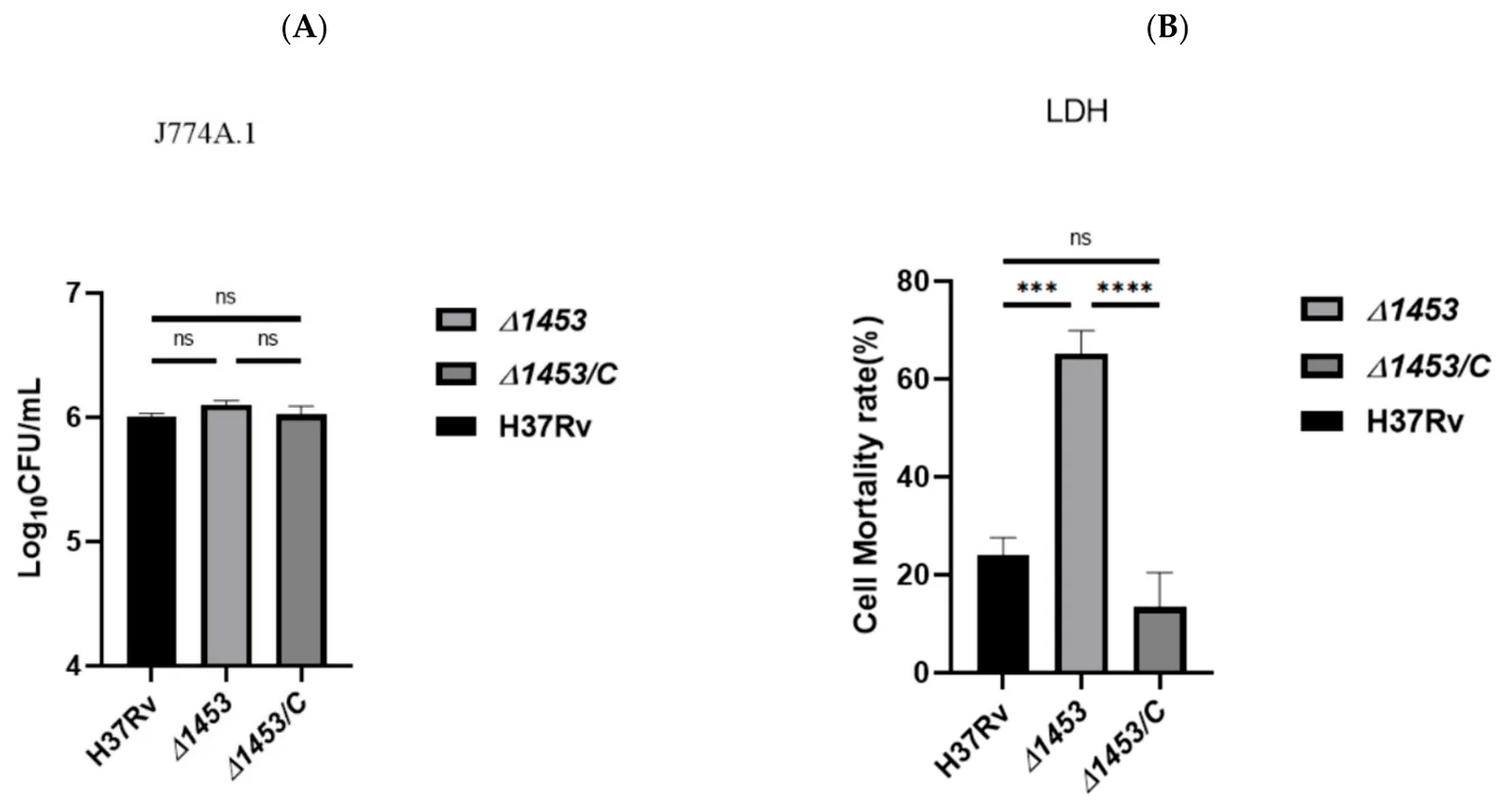
Survival of *Mtb* strains in macrophages. Macrophages were infected with H37Rv, *Δ1453*, and *Δ1453/C* strains at a MOI of 1:1. CFUs of *Mtb* after infection into macrophages (**A**), percentage of LDH release from infected macrophages (**B**). Data are the means ± standard errors from three experiments. *** *p* < 0.001, **** *p* < 0.0001, statistical significance was determined using a one-way ANOVA test.

The survivability of the H37Rv, *Δ1453*, and *Δ1453/C* strains was evaluated by quantifying the bacterial CFUs within macrophages. There was no statistically significant difference in the survivability of the *Δ1453* strain compared to the H37Rv strain within macrophages (Figure 7A). Furthermore, macrophages infected with the *Δ1453* strain exhibited significantly higher levels of LDH release compared to those infected with the H37Rv or *Δ1453/C* strains (Figure 7B). Increased LDH release in infected macrophages points to elevated host-cell cytotoxicity upon *Δ1453* infection.

## 4. Discussion

Transcription factors play a crucial role in conferring reduced multidrug susceptibility by regulating the expression of target genes, thereby influencing changes in *Mtb* efflux [10], metabolism [22], and other processes. In previous research, we demonstrated that Rv1453 suppresses the expression of the *qor* gene [11,12]. However, the mechanisms underlying reduced multidrug susceptibility in the *Δ1453* strain and the specific role of the transcription factor Rv1453 in *Mtb* remain unclear. In this study, we employed transcriptomics, EMSA, and other techniques to investigate the target genes of Rv1453, as well as the molecular mechanisms contributing to reduced multidrug susceptibility in the *Δ1453* strain. Our findings reveal that Rv1453 serves as a key regulator of fatty acid metabolism in *Mtb*, and contributes to multidrug sensitivity.

The thick, lipophilic cell wall of *Mtb* acts as a critical physical barrier, protecting the bacterium from the hostile, bactericidal environment within macrophages and from antibiotics, thus complicating TB treatment [23,24,25]. Impairments in lipid synthesis can compromise the integrity of the *Mtb* cell wall, thereby increasing susceptibility to various anti-tuberculosis drugs [26,27].

The MtrAB two-component system modulates the activity of dihydrolipoamide dehydrogenase (Lpdb) to confer intrinsic reduced multidrug susceptibility, regulate cytokinesis, and maintain cell wall homeostasis in *Mtb* [28]. Correspondingly, research has demonstrated that cell wall thickening frequently occurs under anaerobic, low-oxygen conditions and in response to drug-induced pressure, contributing to the emergence of drug-resistant phenotypes [29]. The cell wall is composed of a complex array of (glyco) lipids, polysaccharides, and peptidoglycans, which collectively reduce drug permeability into the mycobacterial cell [30,31,32,33,34]. MAs are critical components of the *Mtb* cell wall structure. In *Mtb*, MAs can be esterified to the cell wall or exist in a non-bound, solvent-extractable form, including trehalose monomycolate (TMM), trehalose dimycolate (TDM), glucose monomycolate (GMM), and glycerol monomycolate (GroMM) [35]. An increase in the synthesis of fatty acids such as MAs can lead to alterations in the cell wall’s structure and thickness, thereby influencing its permeability to lipophilic drugs. Moreover, the thickening of the cell wall can lead to the relative shortening of pore proteins, thereby influencing the permeability of hydrophilic drugs. The permeability of the *Mtb* cell wall to various drugs is contingent upon a range of drug properties, including physicochemical characteristics and drug morphology. Currently, numerous studies are being conducted to develop models that predict drug permeation through the cell wall [36]. In this context, Rv1453 was involved in transcriptional modulation of lipid metabolism. Changes in mycolic acids (MAs) biosynthesis could modify cell wall characteristics, which may further influence mycobacterial susceptibility to anti-TB drugs.

In terms of fatty acid β-oxidation, there are 36 acyl-CoA synthases (*fadD 1-36*) that catalyze the initial step in fatty acid degradation. The *fadE 1-36* gene encodes acyl-CoA dehydrogenase (ACD), which plays a crucial role by introducing unsaturation into fatty acids. Additionally, there are 21 homologous enzymes within the enoyl-CoA hydratase/isomerase superfamily (*echA 1-21*) that rehydrate the nascent product of the acyl-CoA dehydrogenase reaction. Following successive rounds of β-oxidation, exogenous fatty acids are metabolized to produce acetyl-CoA. Propionyl-CoA can then be condensed with oxaloacetate, resulting in the formation of succinate and pyruvate, which are integral to central metabolism. Moreover, these processes facilitate the synthesis of lipids, particularly cell wall lipids, through the action of Acetyl-CoA carboxylase, encoded by the *accA* and *accD* genes [37]. Consequently, the regulation of lipid metabolism induces alterations in the cell wall’s composition, affecting its thickness, permeability, and the overall morphology of the colony. The genes *fadD18*, *fadE5*, *fadE12*, *fadE13*, *fadE18*, *fadE30*, *fadE31*, *echA7*, *accA2*, and *accD2* have been identified as enriched in the critical steps of the lipid metabolism pathway. We hypothesize that this enrichment enhances the synthesis of fatty acids via β-oxidation and the production of cell wall lipid components in the *Δ1453* strain, ultimately leading to changes in the strain’s lipid composition. However, further experimental verification is required to substantiate these findings.

Sodium valerate, an odd chain fatty acid, undergoes degradation into acetyl-CoA and propionyl-CoA [38]. Substantial accumulation of propionyl-CoA has been shown to be toxic to bacteria. However, this metabolite can be integrated into methyl-branched lipids, such as phthiocerol dimycocerosate, SL-1, polyacyltrehalose, and DAT, which are integral components of the mycobacterial cell wall [39]. Both the H37Rv and *Δ1453/C* strains demonstrated a reduction in CFUs over time when cultured in a medium where sodium valerate serves as the sole carbon source, indicating partial bacterial mortality. Nevertheless, the *Δ1453* strain exhibited enhanced growth, suggesting an improved capacity to metabolize sodium valerate and detoxify the intermediate metabolite propionyl-CoA. The transcriptional up-regulation of *accA2* and *accD2* may drive increased flux toward methyl-branched lipid biosynthesis. As components of acetyl-CoA carboxylase, AccA2-AccD2 complex channels excess propionyl-CoA into the production of methyl-branched lipids. This metabolic sink functions as a detoxification mechanism, as follows: diverting toxic propionyl-CoA away from cytoplasmic accumulation by incorporating it into cell-associated lipid pools. Such rerouting of propionyl-CoA metabolism could mitigate the cytotoxic effects triggered by short- and odd-chain fatty acids. It should be noted that other downstream metabolic enzymes may also participate in this process; further investigation will be required to dissect individual contributions of additional enzymes to propionyl-CoA detoxification. During *Mtb* infection, fatty acids and cholesterol have been identified as the preferred carbon sources for mycobacteria [40]. The transcriptional profiles and lipidomic analyses of *Mtb* within the host environment indicate an up-regulation in the biosynthesis of methylbranched fatty acids, including sulfolipids, DAT, and polyacyltrehalose. This enhancement serves to mitigate the accumulation of toxic propionyl-CoA byproducts, which result from the heightened fatty acid and cholesterol metabolism [41]. Therefore, when the *Δ1453* strain is cultured in a medium where sodium valerate is the sole carbon source, it is likely to produce methyl-branched fatty acids by utilizing increased levels of propionyl-CoA.

The Rv1453 functions as a transcriptional repressor, specifically targeting the promoters of gene clusters involved in lipid metabolism. This regulatory mechanism is analogous to those of transcription regulators such as FadR, YisA, and PsrA, which have been documented in *Sulfolobus acidocaldarius* [42], *Bacillus subtilis* [43], and *Pseudomonas aeruginosa*, respectively. Rv1453 is capable of modulating the expression of various lipid metabolism genes, although its DNA-binding affinity can be inhibited by acyl-CoA [42]. Moreover, the inhibitory effect of C18-acyl-CoA surpasses that of C10-acyl-CoA, indicating a significant role for Rv1453 in the synthesis of long-chain fatty acids. Although the mechanism by which long-chain acyl-CoA inhibits Rv1453 remains incompletely understood, recent research [44] has elucidated the crystal structures of *Mtb* FasR in complex with acyl effector ligands and DNA. These findings indicate that long-chain acyl-CoA modulates protein-DNA binding by regulating the opening of the DNA-binding domain.

Additionally, transcriptomic data suggested a similarly high enrichment for pathways involved in siderophore biosynthesis (Supplementary Figure S5). Iron is crucial for *Mtb,* playing a role in essential biological processes such as electronic transport [45]. To obtain iron from the environment, *Mtb* synthesizes siderophores, which are vital for the *in vivo* growth and survival of the pathogen [46]. The genes are organized into two operons, as follows: *mbt-1*, encompassing the *mbtA-mbtJ* genes, which are responsible for synthesizing the core structure of the branched-chain siderophore molecule; and *mbt-2*, comprising the *mbtK-mbtN* genes, which facilitate the incorporation of hydrophobic aliphatic side chains into the siderophore backbone [47]. In this study, the expression levels of nearly all *mbt-1* genes and all *mbt-2* genes were reduced in the *Δ1453* strain compared to the H37Rv strain. Growth curves for the H37Rv, *Δ1453*, and *Δ1453/C* strains were plotted under iron-deficient, normal iron, and iron-rich conditions. The results indicated no significant differences in the growth of these strains across three media with varying iron concentrations, suggesting bacteria possessed sufficient iron reserves to mitigate toxicity and compensate for iron deficiency in the external environment. Furthermore, the decreased expression of the *irtA* and *irtB* genes (which encode the IrtAB cytoplasmic iron transporter [48]) corroborated this conclusion. Our findings suggest that the Rv1453 protein has the ability to bind to the promoter regions of target genes by recognizing specific motif sequences, including those with high GC content. This implies a potentially extensive regulatory role for the Rv1453 protein. Consequently, further investigation is warranted to ascertain whether genes related to iron metabolism are indeed targets of the Rv1453.

**Figure 8:**
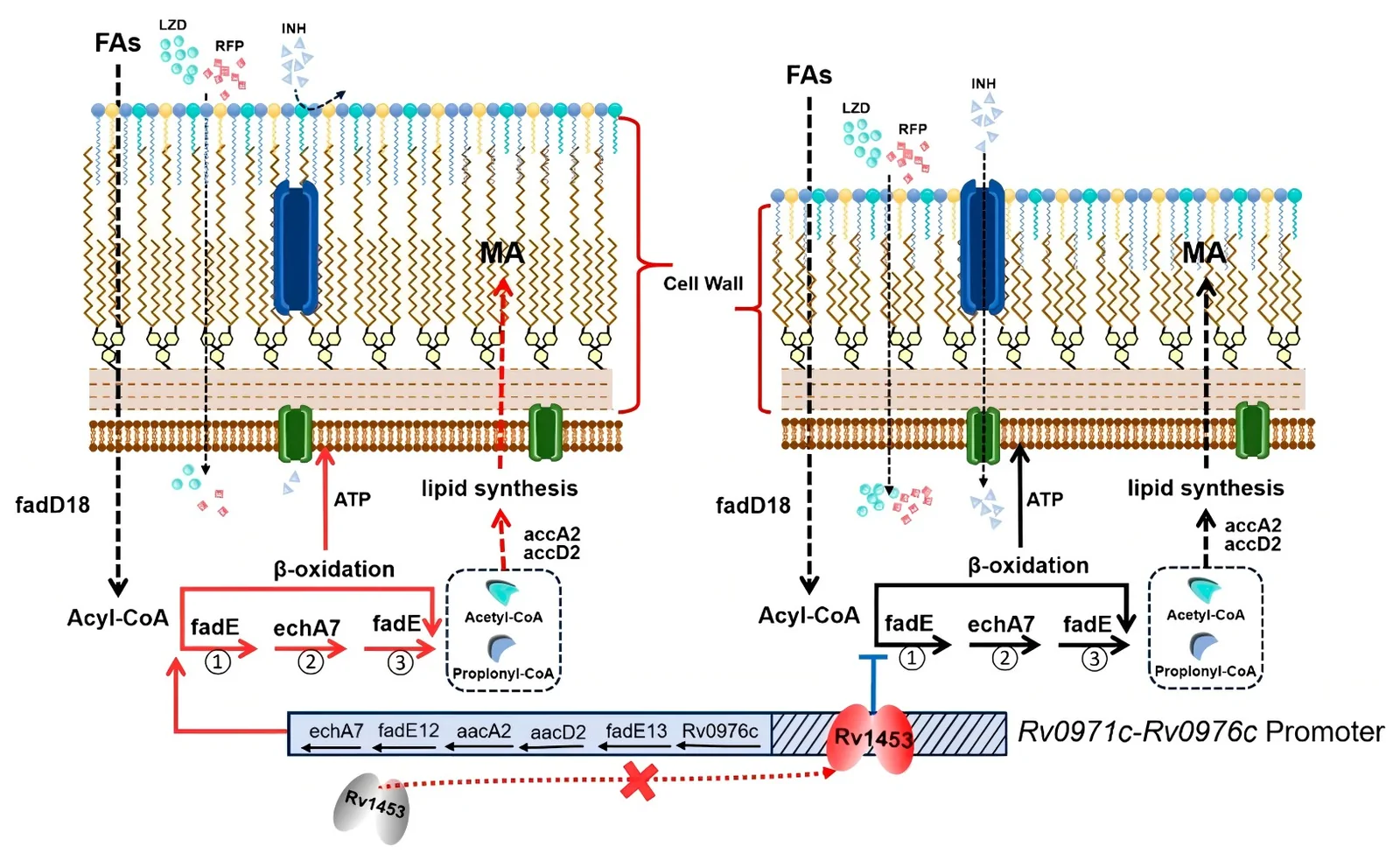
Lipid homeostasis regulated by Rv1453 in *Mycobacterium tuberculosis*. The Rv1453 deletion removes the transcriptional inhibition of fatty acid β-oxidation and lipid syntheses-related enzymes, which results in more exogenous fatty acids being degraded into acetyl-CoA and propionyl-CoA. Acetyl-CoA be used as a precursor for de novo synthesis of fatty acids, also enter the TCA cycle to supply energy to bacteria. Propionyl-CoA is converted to methylmalonyl-CoA by propionyl-CoA carboxylase and methylmalonyl-CoA mutase and used as a precursor for methyl-branched fatty acid synthesis, synthetic cell wall lipids. Ultimately, this impacts bacterial growth, cell wall, and susceptibility to antituberculous drugs. Red color indicates up-regulated and blue color indicates down-regulated. LZD, linezolid; RFP, rifampicin; INH, isoniazid; FAs, fatty acids; MA, mycolic acid; ATP, adenosine triphosphate.

Rv1453 is identified as a master regulator of lipid metabolism genes, as evidenced by knockout bacterial models that exhibit alterations in colonial morphology, cell wall lipid synthesis, and drug sensitivity. In conjunction with previous findings, our data support the model presented in Figure 8, demonstrating that the deletion of Rv1453 affects mycobacterial morphology, cell wall integrity, and antibiotic resistance. The deletion of Rv1453 alleviates transcriptional inhibition of enzymes related to fatty acid β-oxidation and lipid synthesis, leading to increased degradation of exogenous fatty acids into acetyl-CoA and propionyl-CoA. Acetyl-CoA can serve as a precursor for the de novo synthesis of fatty acids via FAS-I, which subsequently contributes to FAS-II induced MAs biosynthesis, and can also enter the TCA cycle to provide energy to bacteria. Propionyl-CoA is enzymatically converted to methylmalonyl-CoA through the actions of propionyl-CoA carboxylase and methylmalonyl-CoA mutase, serving as a precursor for the synthesis of methyl-branched fatty acids and synthetic cell wall lipids. This biochemical pathway ultimately influences bacterial growth, cell wall integrity, and susceptibility to anti-TB drugs. Our study reveals a potential association between Rv1453 deletion-mediated transcriptional regulation of lipid metabolism and reduced multidrug susceptibility in *Mtb*. Rv1453 could potentially be considered as a candidate for further therapeutic investigation. Notably, however, loss of function of Rv1453 confers reduced drug susceptibility, which complicates simple translation into anti-mycobacterial therapies. The development of small molecules that alter the gene-regulatory functions of specific transcription factors—by modulating their interactions with cofactors or DNA, or by disrupting transcription factor dimerization—represents an emerging and promising avenue in drug discovery. Further mechanistic work will be required to evaluate whether targeting Rv1453 can yield beneficial therapeutic outcomes. Furthermore, these observed phenotypes may provide a foundation for future exploration of potential biomarker candidates in subsequent studies.

Despite the mechanistic and phenotypic findings presented in this study, several limitations should be acknowledged. First, although we characterized the phenotypic changes caused by Rv1453 deletion, we did not perform pathway-specific rescue experiments to definitively validate the downstream regulatory network. Second, several functional assays in this work were performed with a limited number of biological replicates, which may restrict the statistical robustness and generalizability of the observed phenotypes. Third, this study relied on the J774A.1 macrophage cell line as an *in vitro* infection model. Although this cell line is widely used for mycobacterial infection studies, it cannot fully recapitulate the complexity of primary macrophages or *in vivo* host immune environments. Fourth, our current EMSA assays have technical caveats, as follows: future experiments using non-specific competitor DNA, nuclease-free purified protein are needed to further confirm the DNA-binding property. Finally, direct structural evidence from circular-dichroism (CD) spectroscopy is lacking to verify proper folding of the refolded recombinant protein. Future work will employ CD spectroscopy to evaluate secondary-structure features of this recombinant protein. Taken together, future pathway-specific rescue experiments, increased biological replicates, improved EMSA experimental conditions, CD-based protein-folding validation, and animal models will help further verify and extend our current conclusions.

## Figures and Tables

**Table 1:** MIC values of various anti-tuberculosis drugs for different strains of *Mtb*.

Strain	MIC (μg/mL)						
	LFX	MFX	EMB	LZD	PA-824	DLM	BDQ
H37Rv	0.292	0.077	3.125	0.535	0.151	0.01	0.039
*Δ1453*	3.984	0.367	25	>25	>5	>0.078	0.078
*Δ1453/C*	0.284	0.106	3.125	0.463	0.156	0.01	0.039

Note: LFX, levofloxacin; MFX, moxifloxacin; EMB, ethambutol; LZD, linezolid; DLM, delamanid; BDQ, bedaquiline.

**Table 2:** Differential lipid and mycobacterial lipid database comparison of *Δ1453*/H37Rv.

*Δ1453*/H37Rv	Number of Differentially Expressed Lipids	Ionic Mode	Numbers	Number of Substances Matched with the Mycobacterial Database
Increased abundance	639	POS	502	90
		NEG	137	22
Decreased abundance	477	POS	383	81
		NEG	94	27

## Data Availability

The transcriptomics data have been deposited in NCBI and are available under the accession number PRJNA1309890.

## Supplementary Materials

The following supporting information can be downloaded at: https://www.mdpi.com/article/10.3390/microorganisms14102376/s1, Table S1: MIC values of various anti-tuberculosis drugs for different strains of *Mtb*; Figure S1: GO assays and KEGG pathway assays for the genes that are differentially expressed in *Δ1453* strains; Figure S2: *fadE12*, *fadE13*, *accA2*, *accD2* and *echA7* genes are parts of the same operon; Figure S3: Full-length gels and blots; Figure S4: TEM observation of the cell walls of each strain; Figure S5: Heat maps illustrating the log2FC values of genes related to siderophore biosynthesis pathway in H37Rv and *Δ1453* strains; Figure S6: SDS-PAGE analysis of Rv1453 protein.
