# Supplementary material for: Transcription Factor Rv1453 Contributes to Multidrug Sensitivity of Mycobacterium tuberculosis: A Potential Role for Lipid Metabolism
Source: Microorganisms. 2026 Oct 16;14(10):2376. doi: 10.3390/microorganisms14102376 (PMC13707636; doi:10.3390/microorganisms14102376)
Supplement: Supplementary file 1 [file microorganisms-14-02376-s001.zip › Supplementary data.pdf]

Supplementary data

Table S1. MIC values of various anti-tuberculosis drugs for different strains of *Mtb*

Strain	MIC（μg/mL）			
	INH	RFP	LFX	BDQ
H37Rv	0.039	0.02	0.313	0.039
qor overexpression	0.039	＜0.01	0.078	＜0.01

Note: INH, isoniazid; RFP, rifampicin; LFX, levofloxacin; BDQ, bedaquiline.

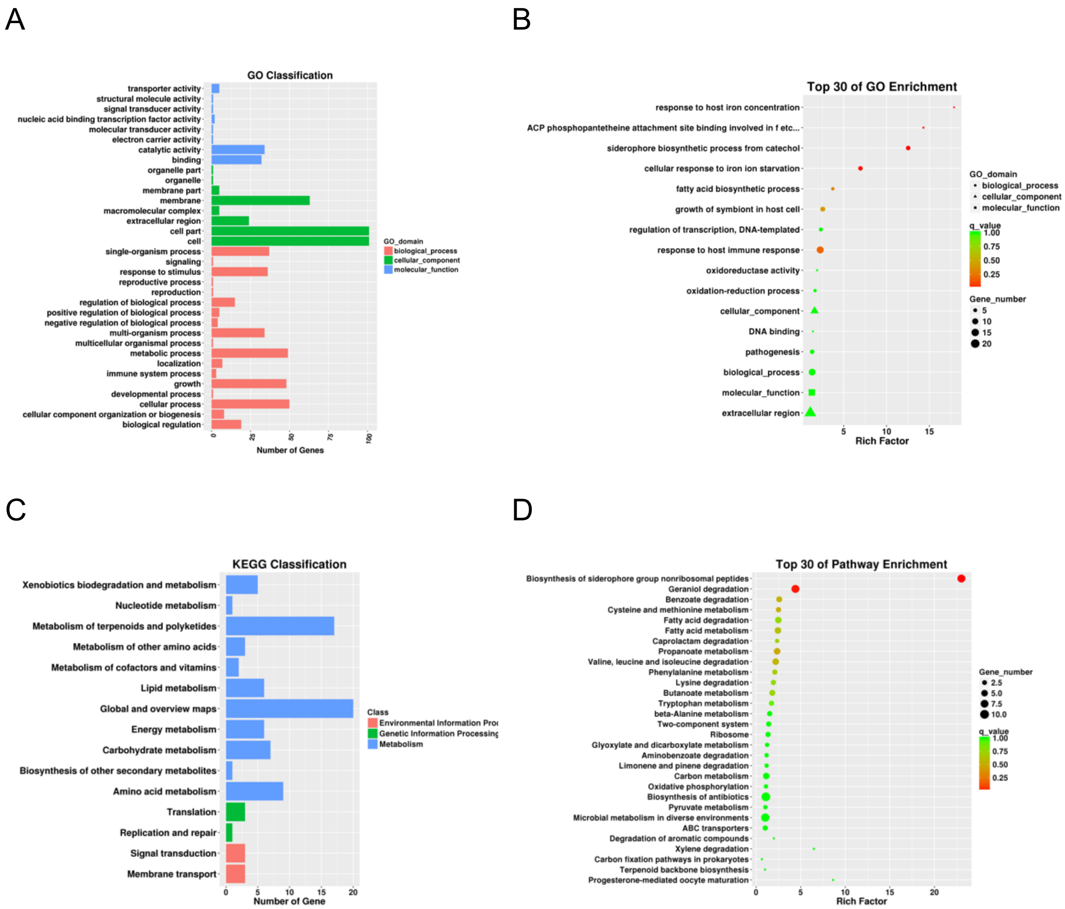

Figure S1. GO assays and KEGG pathway assays for the genes that are differentially expressed in *Δ1453* strains.

A, B: GO assays for the genes that are differentially expressed in *Δ1453* strains, n=3;

C, D: KEGG pathway assays for the genes that are differentially expressed in *Δ1453* strains, n=3.

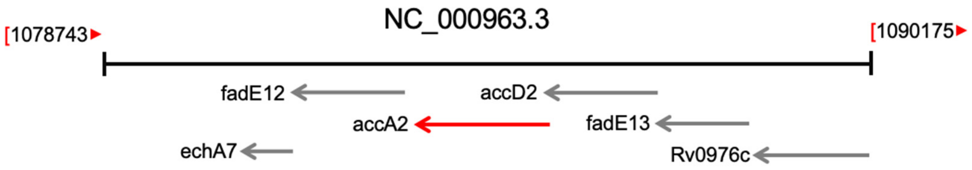

Figure S2. *fadE12*, *fadE13*, *accA2*, *accD2* and *echA7* genes are parts of the same operon.

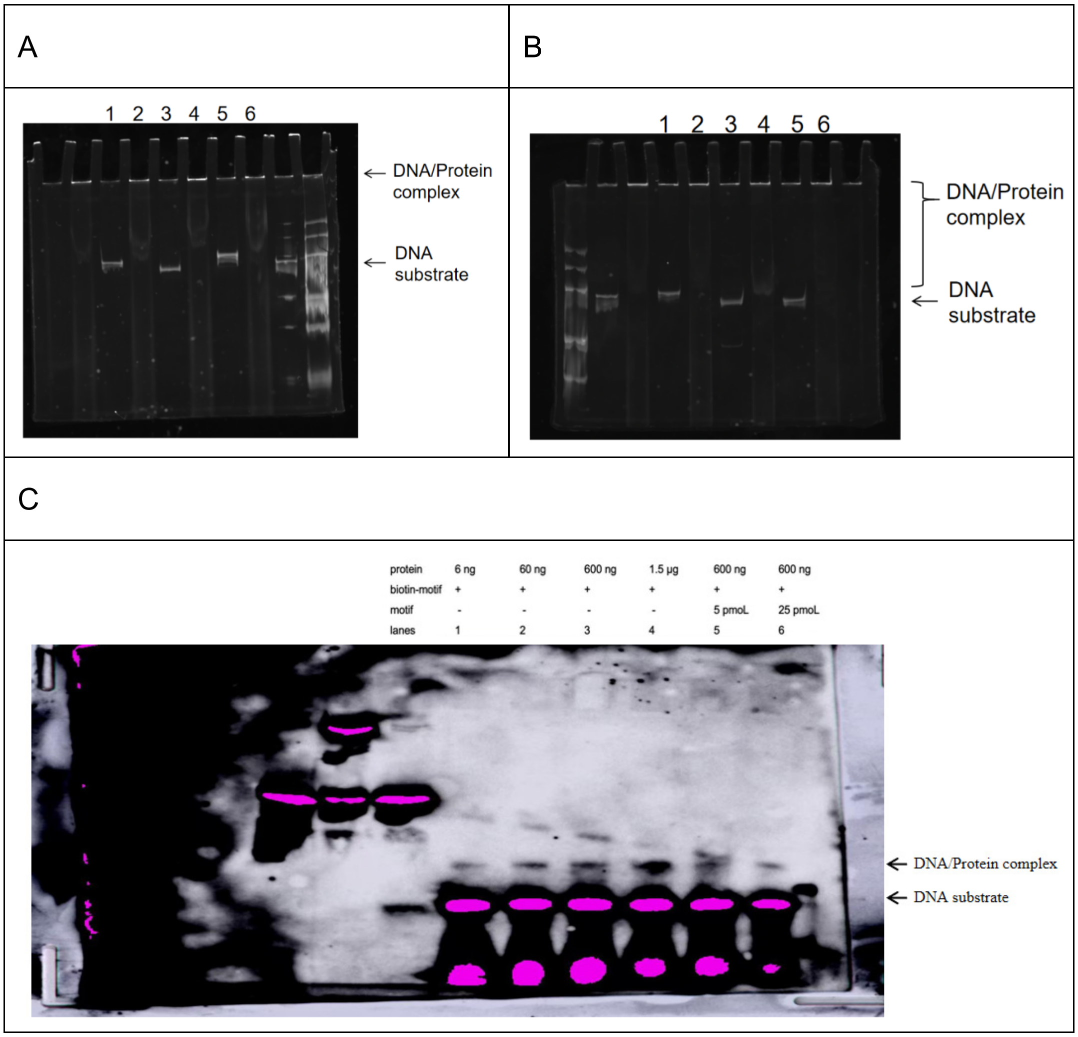

Figure S3. Full-length gels and blots

A: Full-length gel and blot of figure 2A. EMSA assays for DNA-binding activity of Rv1453 with the promoters of the gene operon (*fadE18p*, *fadE5p*, *fadD18p*). 1: *fadE18p*; 2: *fadE18p* + Rv1453; 3: *fadE5p*; 4: *fadE5p* + Rv1453; 5: *fadD18p*; 6: *fadD18p* + Rv1453.

B: Full-length gel and blot of figure 2B. EMSA assays for DNA-binding activity of Rv1453 with the promoters of the gene operon (*Rv0976cp*, *fadE31p*, *fadE30p*). 1: *Rv0976cp*; 2: *Rv0976cp* + Rv1453; 3: *fadE31p*; 4: *fadE31p* + Rv1453; 5: *fadE30p*; 6: *fadE30p* + Rv1453.

C: Full-length gel and blot of figure 3B. Competitive EMSA to determine the binding of Rv1453 to the motif sequence.

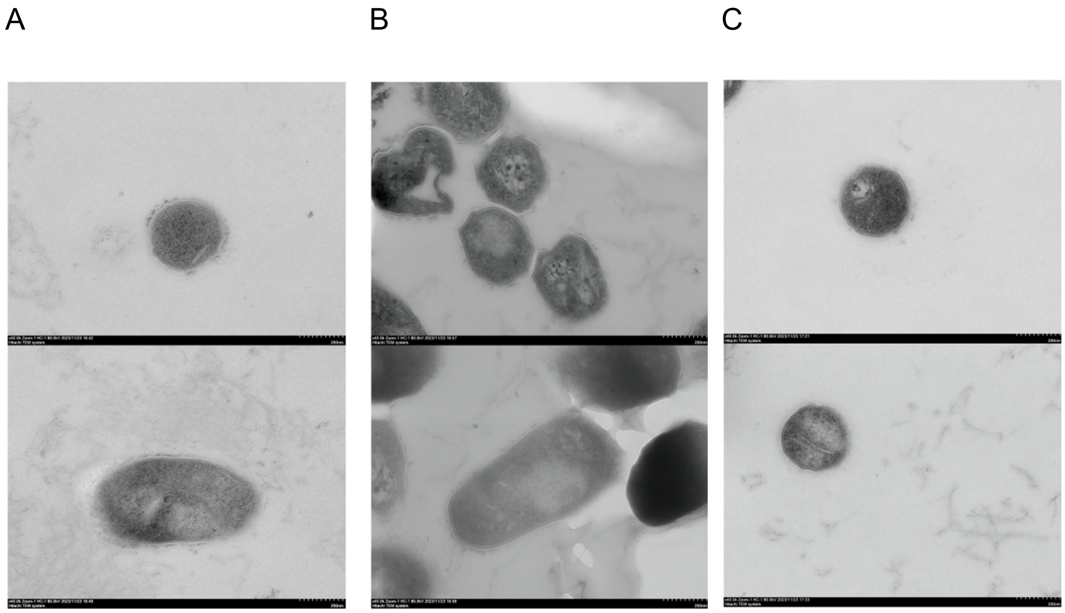

Figure S4. TEM observation of the cell walls of each strain. A: H37Rv; B: *Δ1453*; C: *Δ1453/C*.

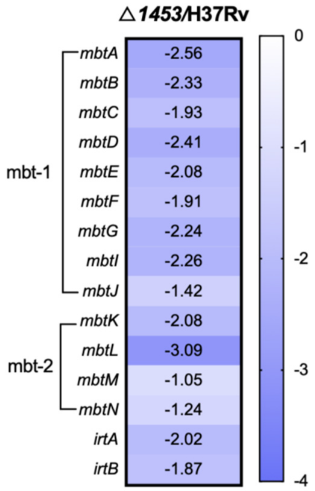

Figure S5. Heat maps illustrating the log2FC values of genes related to siderophore biosynthesis pathway in H37Rv and *Δ1453* strains. Note: Color intensity shows relative up- or down regulation.

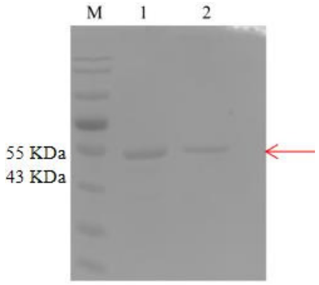

Figure S6. SDS-PAGE analysis of Rv1453 protein
